# Phenotype Expansion for Atypical Gaucher Disease Due to Homozygous Missense PSAP Variant in a Large Consanguineous Pakistani Family

**DOI:** 10.3390/genes13040662

**Published:** 2022-04-09

**Authors:** Khurram Liaqat, Shabir Hussain, Anushree Acharya, Abdul Nasir, Thashi Bharadwaj, Muhammad Ansar, Sulman Basit, Isabelle Schrauwen, Wasim Ahmad, Suzanne M. Leal

**Affiliations:** 1 Center for Statistical Genetics, Gertrude H. Sergievsky Center, The Department of Neurology, Columbia University Medical Center, New York, NY 10032, USA; kl3353@cumc.columbia.edu (K.L.); aa4471@cumc.columbia.edu (A.A.); tb2890@cumc.columbia.edu (T.B.); is2632@cumc.columbia.edu (I.S.); 2 Department of Biotechnology, Faculty of Biological Sciences, Quaid-i-Azam University, Islamabad 45320, Pakistan; 3 Department of Biochemistry, Faculty of Biological Sciences, Quaid-i-Azam University, Islamabad 45320, Pakistan; shabbirhussain313@gmail.com (S.H.); ansar@qau.edu.pk (M.A.); wahmadqau@gmail.com (W.A.); 4 Synthetic Protein Engineering Lab (SPEL), Department of Molecular Science and Technology, Ajou University, Suwon 443-749, Korea; anasirqau@gmail.com; 5 Center for Genetics and Inherited Diseases, Taibah University, Almadinah Almunawarah, Medina 42318, Saudi Arabia; sbasit.phd@gmail.com; 6 Taub Institute for Alzheimer’s Disease and the Aging Brain, Columbia University Medical Center, New York, NY 10032, USA

**Keywords:** atypical Gaucher disease, hearing impairment, exome sequencing, saposin C

## Abstract

Atypical Gaucher disease is caused by variants in the *PSAP* gene. Saposin C is one of four homologous proteins derived from sequential cleavage of the saposin precursor protein, prosaposin. It is an essential activator for glucocerebrosidase, which is deficient in Gaucher disease. Although atypical Gaucher disease due to deficiency of saposin C is rare, it exhibits vast phenotypic heterogeneity. Here, we report on a Pakistani family that exhibits features of Gaucher disease, i.e., prelingual profound sensorineural hearing impairment, vestibular dysfunction, hepatosplenomegaly, kyphosis, and thrombocytopenia. The family was investigated using exome and Sanger sequencing. A homozygous missense variant c.1076A>C: p.(Glu359Ala) in exon 10 of the *PSAP* gene was observed in all affected family members. In conclusion, we identified a new likely pathogenic missense variant in *PSAP* in a large consanguineous Pakistani family with atypical Gaucher disease. Gaucher disease due to a deficiency of saposin C has not been previously reported within the Pakistani population. Genetic screening of patients with the aforementioned phenotypes could ensure adequate follow-up and the prevention of further complications. Our finding expands the genetic and phenotypic spectrum of atypical Gaucher disease due to a saposin C deficiency.

## 1. Introduction

Gaucher disease (GD) is a lysosomal storage disease with an autosomal recessive mode of inheritance. The disease phenotype occurs due to accumulation of glucosylceramide mainly in lysosomes of cells of the monocyte/macrophage system. The deficiency of the lysosomal enzyme glucocerebrosidase (GCase) is known to cause GD. Based on differences in the clinical features, GD is divided into three types: type 1 (GD1; MIM 230800), type 2 (GD2; MIM 230900), and type 3 (GD3; MIM 231000). These clinical subtypes are defined based on the severity of manifestations, age of onset, and neurological involvement [1]. GD1 is non-neuronopathic, GD2 is an acute neuronopathic form, and GD3 presents with slowly progressive neurological involvement [2]. The β-glucocerebrosidase enzyme is a lysosomal enzyme that belongs to the glycoside hydrolase family encoded by *GBA* (MIM 606463) [3]. The common features associated with GD include hepatosplenomegaly, anemia, thrombocytopenia, hearing impairment (HI), and osteopenia. Prosaposin is another lysosomal protein, encoded by *PSAP* (MIM 176801), which undergoes post-translational cleavage to yield four proteins named saposins (Sap) A, B, C, and D. Variants in *PSAP* that cause a Sap-C deficiency are known to underlie atypical GD (MIM 610539) and can resemble either GD1 or GD3 [4]. Sap-C acts as an activator of GCase enzyme that is required for glucosylceramide degradation. Deficiency of Sap-A due to *PSAP* variants causes atypical Krabbe disease (MIM 611722) and inadequate production of Sap-B leads to metachromatic leukodystrophy (MIM 249900). Sap-D deficiency has been studied in a mouse model and causes ceramide lipidosis that resembles human Farber disease [5]. Although most cases of GD are due to variants within the *GBA* gene that codes for GCase, seven patients with variants in the *PSAP* gene that encodes for Sap-C have been previously described (Table 1).

In this report, we present the clinical and genetic characterization of a Pakistani family with atypical GD caused by a likely pathogenic homozygous variant in *PSAP*. This is the first report in the Pakistani population of atypical GD due to a variant in *PSAP*.

## 2. Methods

### 2.1. Family History and Clinical Evaluation

This study was approved by the Institutional Review Boards of Quaid-i-Azam University (QAU-153) and Columbia University, New York (AAAS3433). A consanguineous family, DEM4599, with syndromic HI was ascertained from a rural area of Sindh Province in Pakistan. Written informed consent was obtained from all participating family members.

**Figure 1:**
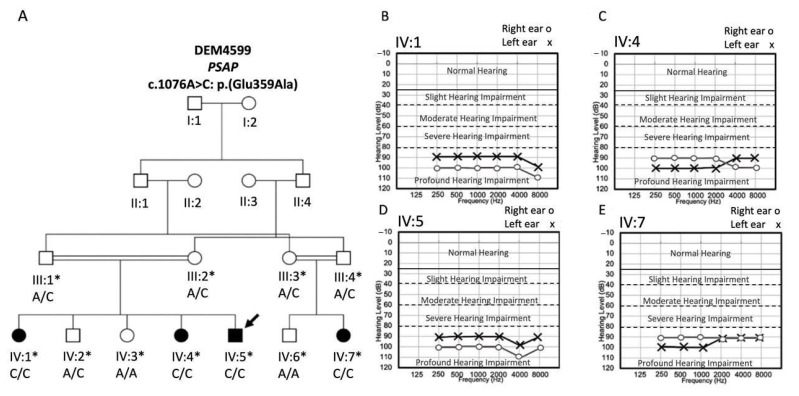
Pedigree and audiograms for family DEM4599. (**A**) Pedigree drawing of family DEM4599 segregating GD with an autosomal recessive mode of inheritance. Circles represent females and squares males. Filled symbols indicate affected family members and clear symbols unaffected members. Double lines in the pedigree represent a consanguineous marriage. Asterisks indicate individuals for whom a DNA sample is available, and an arrow indicates the sample selected for exome sequencing. Below each family member the *PSAP* c.1076A>C genotype is displayed. Audiograms of the affected individuals of the family DEM4599 (**B**) IV:1 (12 years of age), (**C**) IV:4 (14 years of age), (**D**) IV:5 (20 years of age), and (**E**) IV:7 (19 years of age).

Family DEM4599 displays an autosomal recessive mode of inheritance for atypical GD (Figure 1A). Blood samples were obtained from four affected (IV:1, IV:4, IV:5, and IV:7) and seven unaffected (III:1, III:2, III:3, III:4, IV:2, IV:3, and IV:6) family members. Affected individuals were between 12 and 24 years of age at the time of ascertainment. Genomic DNA was extracted from peripheral blood using a phenol chloroform procedure [11]. Pure tone audiometry, abdominal ultrasound, and complete blood count (CBC) blood test were performed for all affected family members at a local government hospital. Affected family members also underwent tandem gait and Romberg tests to determine if they had any vestibular dysfunction. For affected individual IV:5 an X-ray of the vertebral column, and a computerized tomography (CT) scan were also performed.

### 2.2. Exome Sequencing and Bioinformatics Analysis

Exome sequencing was performed using a DNA sample from affected member IV:5. For the preparation of exome libraries, the SureSelect Human All Exon V6 kit (60.46 Mb target region) was used. Sequencing was performed using 100 bp paired-end on a HiSeq2500/4000 instrument (Illumina Inc, San Diego, CA, USA). The Burrows-Wheeler Aligner-MEM (BWA) was used to align the reads with the human reference genome (GRCh37/Hg19) [12]. Picard-tools was used to perform duplicate marking. Single nucleotide variants and insertions/deletions (InDels) were jointly called using the genome analysis toolkit (GATK) [13].

Conservation and bioinformatic predictions of the variants were annotated in silico using ANNOVAR and dbNSFP [14,15]. Filtering was performed to further analyze the exome sequence data. Frameshift, in-frame inDels, missense start/stop altering, nonsense, and splice-site variants with a minor allele frequency < 0.005 in every population of the Genome Aggregation Database (gnomAD) [16] population that were either homozygous or potentially compound heterozygous were retained. 

DNA samples from all family members were sequenced using Sanger sequencing on an ABI 3130 Genetic Analyzer (Applied Biosystems, Foster City, CA, USA) to verify segregation of the identified variant. Primers surrounding the region of interest were designed using the primer3 software [17]. The identified variant that segregated with atypical GD was classified according to the American College of Medical Genetics and Genomics (ACMG) guidelines [18,19].

### 2.3. Linkage Analysis 

Two-point parametric linkage was performed using MLINK of the LINKAGE software package [20]. Genotypes obtained from Sanger sequencing for the identified variant were analyzed. Linkage analysis was performed using an autosomal recessive mode of inheritance with complete penetrance, no-phenocopies, and a disease and variant allele frequency of 3.6 × 10^−4^, which is the frequency for the identified variant for South Asians in gnomAD.

### 2.4. Three-Dimensional Protein Modeling

The crystal structure of human Sap-C (PDB ID: 2GTG) was obtained from the Protein Data Bank (PDB) [21]. The three-dimensional modeling program, MODELLER, was used to build wild-type and mutant PSAP (p.Glu359Ala) structures [22]. PYMOL was used for structural visualization and image processing (The PyMOL Molecular Graphics System, Version 1.8 Schrödinger, LLC).

## 3. Results

### 3.1. Clinical Description

Pure-tone audiometry diagnosed bilateral profound HI in all DEM4599 affected pedigree members IV:1, IV:4, IV:5, and IV:7 according to World Health Organization classification (Figure 1B–E). The HI was reported to be prelingual and most likely congenital. All affected individuals (IV:1, IV:4, IV:5, and IV:7) showed vestibular dysfunction evaluated via tandem gait and Romberg tests. Affected study participants were unable to complete the tandem gait test without swaying and losing their balance and they also could not maintain their balance during the Romberg test. Additionally, problems with balance were observed since early childhood for the affected family members. The whole abdomen ultrasound for each affected family member revealed hepatosplenomegaly, however the kidneys and pancreas were normal. Liver and spleen measurements of affected individuals are given in Table 2. All affected family members had thrombocytopenia. Myopia was observed in affected individuals IV:1 and IV:5, while affected family members IV:4 and IV:7 had normal vision. A CT scan of affected individual IV:5, revealed a normal middle and inner ear with intact cochleae, vestibules, and semicircular canals. No brain abnormalities were observed. Other than vestibular dysfunction, no neurological or behavioral abnormalities were observed for the affected family members. X-rays of the vertebral column of affected individual IV:5 displayed kyphosis (Supplementary Figure S1). None of the affected individuals showed abnormal ECG waveform. The height and weight of each affected individual were measured and showed normal body growth (Table 2). Furthermore, additional symptoms such as epilepsy/seizures, intellectual impairment, and failure to thrive which are seen in other GD patients were not observed in the affected family members. Clinical parameters for affected family members of DEM4599 are displayed in Table 2. 

### 3.2. Exome and Sanger Sequencing Results

**Figure 2:**
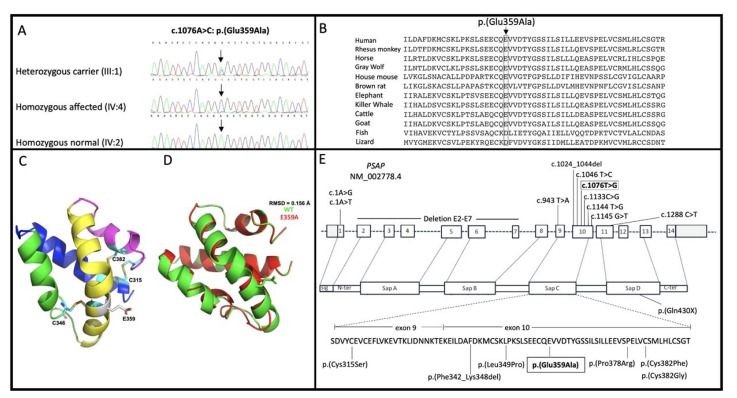
Overview of PSAP (NM_002778.4) protein domains, sequence data on variant c.1076A>C, p.(Glu359Ala) in family DEM4599 and a predicted three-dimensional structure of Sap-C. (**A**) Chromatograms showing likely pathogenic missense variant c.1076A>C, p.(Glu359Ala) in the *PSAP* in heterozygous unaffected carrier (III:1) (upper panel), homozygous affected family member (IV:4) (middle panel), and homozygous wild-type unaffected family member individual (IV:2) (bottom panel). (**B**) The mutated amino acid residue Glu359 located in the Sap-C domain is conserved among various species analyzed. (**C**) Three-dimensional structure of the wild-type Sap-C domain in ribbon representation with the conserved cysteine residues are indicated. (**D**) The superposed structure of wild (green color) and mutant (red color) structure showing the difference in overall confirmation due to the p.E359A mutation. RMSD (root mean square deviation). (**E**) Structure of *PSAP* (NM_002778.4) and the protein it encodes. The protein is composed of Sap A-D domains. The missense variant c.1076A>C, p.(Glu359Ala) located in exon 10 of the *PSAP* (NM_002778.4) identified in this study is displayed in a box. All other variants which have been identified for atypical GD are indicated by a line. Exons 9 and 10 of the *PSAP* encode the Sap-C domain. Besides c.1076A>C, p.(Glu359Ala) the only other variant that was previously observed in the Sap-C domain in the homozygous state is p.(Phe342_Lys348del).

Analysis of exome sequence data revealed a homozygous missense variant [c.1076A>C: p.(Glu359Ala)] in *PSAP* (NM_002778.4). No additional rare variants were observed in known HI genes. Sanger sequencing of this variant using DNA samples from all family members participating in the study showed that the variant segregated with atypical GD within the family (Figure 2A). The variant had a LOD score of 3.98 at θ = 0.0.

### 3.3. In Silico Analysis

The variant c.1076A>C: p.(Glu359Ala) is rare with a gnomAD allele frequency (AF) = 5.3 × 10^−5^ in all populations and a South Asian AF = 3.6 × 10^−4^ with no homozygous variants in any gnomAD population. The variant is in the Sap-C domain of the prosaposin protein and is predicted to be damaging by various bioinformatic tools including MutationTaster and Polyphen2 and additionally has a CADD c-score = 28. The amino acid residue Glu359 in PSAP is highly conserved among various species (Figure 2B) and has a GERP++ score = 5.67. The variant was classified as likely pathogenic (PP1-S, PP3, PM2) according to the ACMG guidelines for variant classification.

### 3.4. Three-Dimensional Modeling

The three-dimensional structures of the wild type and mutant PSAP proteins were built using homology modeling techniques to inspect the potential effect of the variant p.Glu359Ala on the Sap domain (Figure 2C). The Glu359 in the wild-type resides at the helix region of the hairpin. The substitution of polar charged glutamate into a small non-polar charged side chain can affect the overall confirmation. The wild-type superposition with p.Glu359Ala displays 0.156 Å RMSD (root mean square deviation), revealing a difference in overall conformation due to the variant in the Sap domain (Figure 2D).

## 4. Discussion

In this study, we present a family with atypical GD, characterized at clinical and molecular level. We identified a missense variant c.1076A>C: p.(Glu359Ala) in exon 10 (NM_002778.4) of *PSAP* in a family with atypical GD. This variant has been previously reported as a variant of uncertain significance in ClinVar (accession number: VCV000991967.1) for metachromatic leukodystrophy, but no classification criteria were specified. 

Atypical GD resulting from Sap-C deficiency was first reported in 1986 [23]. There are only seven cases reported in the literature, of which two are siblings with compound heterozygous variants (p.(Met1Leu)/p.(Leu349Pro)) in exons 1 and 10 of *PSAP*. None of the PSAP variants underlying atypical GD have been observed more than once in unrelated individuals (Table 1). 

*PSAP* variants that cause GD have been reported in families from China, France, India, Poland, Spain, and Sweden (Table 1). Over 350 variations associated with GD in the *GBA* gene have been identified [24] but only ten variants in the *PSAP* gene have been reported to cause atypical GD (Table 1; Figure 2E). Out of these ten variants six (p.(Cys315Ser), p.(Leu349Pro), p.(Pro378Arg), p.(Cys382Phe), p.(Cys382Gly), and p.(Phe342_Lys348del)) are in the Sap-C domain, while the other 4 variants are likely loss-of-function affecting all saposins (Figure 2; Table 1). Four of the variants in the Sap-C domain (p.(Cys315Ser), p.(Phe342_Lys348dell), p.(Cys382Gly), and p.(Cys382Phe)) affect a cystine residue and the patients carrying these variants also had neurological abnormalities [25]. This is probably due to disruption of disulfide bridges [26]. The CT scan failed to reveal any neurological abnormalities in affected family member IV:5. It is likely that the variant p.(Glu359Ala), which we identified is not involved in establishing a disulfide bridge in the protein and therefore there are no neurological abnormalities observed. In contrast to our finding, the neurological abnormalities were observed in some of the previously reported cases with atypical GD due to Sap-C deficiency [27]. 

The Sap-binding site with glucocerebrosidase is located within amino acids 351–390 of PSAP, and a 16-amino acid sequence (357–372) is important for glucocerebrosidase activation [28]. The identified variant in this study Ala359 is also found in the same binding site. We suggest that this variant Ala359 may affect the activation of glucocerebrosidase. Furthermore, 3D modeling shows that the Ala359 causes conformational change in Sap-C in the mutant protein, but it does not alter the disulfide bridge. The Glutamate amino acid at position 359 in PSAP is highly conserved among various species, although in reptiles and fish at position 359, aspartic acid is present. 

In the previously reported GD cases that involved compound heterozygous variants, four missense variants (p.(Cys315Ser), p.(Leu349Pro), p.(Pro378Arg), and p.(Cys382Gly)) were within the Sap-C domain while the four trans variants (p.(Met1Val), p.(Met1Leu), p.(Gln430X), and delE2–E7) were found outside of the Sap-C domain and may abolish the production of all saposins [5]. For one patient a single variant p.(Cys382Phe) was found in the Sap domain and an additional variant was not identified [6]. Previously, only a single homozygous variant p.(Phe342_Lys348del) in the Sap-C domain had been reported [9].

*PSAP* is expressed in the inner ear cells of mice. Furthermore, *PSAP* knockout mice exhibit severe vestibular dysfunction and HI [29]. We also observed the HI and vestibular dysfunction in all the affected family members of DEM4599 that were not observed in previously reported Sap-C deficient GD patients. In addition to the aforementioned phenotypes, affected family members also presented with hepatosplenomegaly, thrombocytopenia, kyphosis. A limitation of this study is that we were unable to assess plasma chitotriosidase or glucosylsphingosine in the affected family members since they live in a remoted region of Pakistan.

In Summary, we report on a new likely pathogenic missense variant (c.1076A>C: p.(Glu359Ala)) in *PSAP* and expand the phenotypic spectrum of atypical GD due to Sap-C deficiency to include vestibular dysfunction and HI. In this study, for the first time, we described the patients with atypical GD due to Sap-C deficiency from the Pakistani population. We recommend genetic testing of individuals displaying features of GD with congenital HI, to ensure adequate follow-up and prevention of clinical complications.

## Figures and Tables

**Table 1:** Overview of Gaucher disease patients with saposin C deficiency.

Patient	Gender	Clinical Features	cDNA Variant (Allele 1/Allele 2)	Protein Variant (Allele 1/Allele 2)	Exon	Origin	Reference
1	Female	Hepatosplenomegaly, Seizure	c.1145G>T/ unknown	p.(Cys382Phe)/ unknown	10	Sweden	[6]
2	Male	Hepatosplenomegaly, Seizure, Ataxia, tremor, ophthalmoplegia	c.1144T>G/ c.1288 C>T	p.(Cys382Gly)/ p.(Gln430X)	10, 11	Spain	[7]
3	Female	Hepatosplenomegaly, intellectual decline, epilepsy	c.1A>G/ c.943T>A	p.(Met1Val)/ p.(Cys315Ser)	1, 9	France	[8]
4	Male	Hepatosplenomegaly, osteopenia	c.1A>T/ c.1046 T>C	p.(Met1Leu)/ p.(Leu349Pro)	1, 10	Poland	[5]
5	Female	Hepatosplenomegaly, osteopenia	c.1A>T/ c.1046T>C	p.(Met1Leu)/ p.(Leu349Pro)	1, 10	Poland	[5]
6	Female	Hepatosplenomegaly	c.1024_1044del/ c.1024_1044del	p.(Phe342_Lys348del)/ p.(Phe342_Lys348del)	10, 10	India (Sikh)	[9]
7	Male	Hepatosplenomegaly, thrombocytopenia, anemia, abnormal electroencephalogram	c.1133C>G/ delE2–E7	p.(Pro378Arg)/nonsense mediated mRNA decay	10, delE2–E7	China	[10]
8	Female IV:1	Hepatosplenomegaly, thrombocytopenia, kyphosis, Myopia (late onset), vestibular dysfunction, hearing impairment	c.1076A>C/ c.1076A>C	p.(Glu359Ala)/ p.(Glu359Ala)	10, 10	Pakistan	Present study
9	Female IV:4	Hepatosplenomegaly, thrombocytopenia, kyphosis, vestibular dysfunction, hearing impairment	c.1076A>C/ c.1076A>C	p.(Glu359Ala)/ p.(Glu359Ala)	10, 10	Pakistan	Present study
10	Male IV:5	Hepatosplenomegaly, thrombocytopenia, kyphosis, Myopia (late onset), vestibular dysfunction, hearing impairment	c.1076A>C/ c.1076A>C	p.(Glu359Ala)/ p.(Glu359Ala)	10, 10	Pakistan	Present study
11	Female IV:7	Hepatosplenomegaly, thrombocytopenia, kyphosis, vestibular dysfunction, hearing impairment	c.1076A>C/ c.1076A>C	p.(Glu359Ala)/ p.(Glu359Ala)	10, 10	Pakistan	Present study

**Table 2:** Clinical parameters of affected individuals of family DEM4599.

Parameters	Individual IV:1	Individual IV:4	Individual IV:5	Individual IV:7
Age (yrs)	12	14	20	19
Sex	Female	Female	Male	Female
Height (cm)	139	145	168	162
Weight (kg)	38	42	69	61
Platelets count/mm^3^	95,000	100,000	88,000	90,000
Liver Size (cm)	15.4	15.8	17.2	16.0
Spleen Size (cm)	13.4	13.7	14	13.9
Hearing Impairment	Profound	Profound	Profound	Profound
Vestibular dysfunction	Yes	Yes	Yes	Yes

## Data Availability

The variant has been deposited in the ClinVar database [Accession number: SCV001652887 (PSAP [c.1076A>C: p.(Glu359Ala)]).

## Supplementary Materials

The following supporting information can be downloaded at: https://www.mdpi.com/article/10.3390/genes13040662/s1, Figure S1: X-rays of vertebral column (A–C) showing kyphosis in an affected family member (IV:5).
