# Supplementary figures and images for: Phenotype Expansion for Atypical Gaucher Disease Due to Homozygous Missense PSAP Variant in a Large Consanguineous Pakistani Family
Source: Genes (Basel). 2022 Apr 9;13(4):662. doi: 10.3390/genes13040662 (PMC9028228; doi:10.3390/genes13040662)

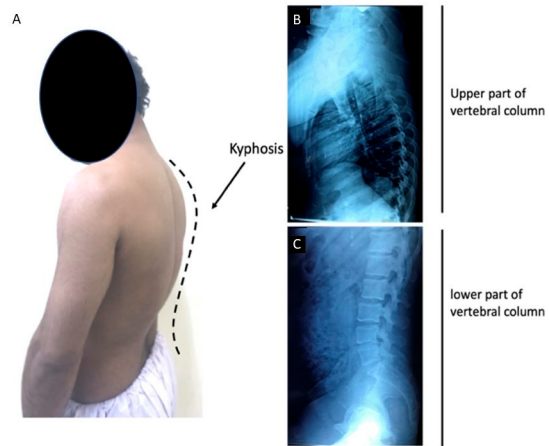

Figure S1: X-rays of vertebral column (A–C) showing kyphosis in an affected family member (IV:5).

Supplement: Supplementary file 1 [file genes-13-00662-s001.zip › genes-1621812-supplementary.pdf]
